# Color Stability, Chemico-Physical and Optical Features of the Most Common PETG and PU Based Orthodontic Aligners for Clear Aligner Therapy

**DOI:** 10.3390/polym14010014

**Published:** 2021-12-21

**Authors:** Valeria Daniele, Ludovico Macera, Giuliana Taglieri, Loredana Spera, Giuseppe Marzo, Vincenzo Quinzi

**Affiliations:** 1 Department of Industrial and Information Engineering and Economics, University of L’Aquila, Piazzale Pontieri 1, Monteluco di Roio, 67100 L’Aquila, Italy; valeria.daniele@univaq.it (V.D.); giuliana.taglieri@univaq.it (G.T.); loredana.spera@univaq.it (L.S.); 2 Department of Life, Health & Environmental Sciences, Postgraduate School of Orthodontics, University of L’Aquila, P.le Salvatore Tommasi 1, Ed. Delta 6, Coppito, 67100 L’Aquila, Italy; giuseppe.marzo@univaq.it (G.M.); vincenzo.quinzi@univaq.it (V.Q.)

**Keywords:** comparison between clear orthodontic aligners, X-ray diffraction, differential scanning calorimetry, optical properties, color stability, scanning electronic microscopy, water absorption, simulated intraoral environment

## Abstract

It is difficult to find research papers collecting comparative results about characterization studies of clear aligners. Therefore, the aim of this paper is to provide the first comparative analysis of most commercial clear aligners, in terms of their stability towards intra-oral staining agents, their physicochemical and optical properties, as well as their water absorption behavior. Five types of aligners, characterized by different techniques, are considered: Erkodur, Essix Plastic, Ghost Aligner, Zendura, and Invisalign. The obtained results show that clear aligners are made up of PETG, semi rigid PU, and a mixture of PU and PETG, with different degrees of crystallinity which affect the transparency of each aligner. In particular, the PETG-based materials reveal the highest value of short-range order and the highest properties in terms of transparency in the visible range. After 14 days of immersion into red wine and coffee, PETG and PU-based aligners reveal a perceivable change in color (NBS values from 1.5 to 3), corresponding to a loss of transparency due to the deposition of impurities on the surface. These results are particularly marked for Invisalign, showing changes towards other colors (NBS up to 35), probably due to the thermoforming process which led to the formation of a wrinkled surface entrapping the impurities.

## 1. Introduction

Since their original development in 1997 with the Invisalign^®^ technology [1], clear thermoformed plastic aligners are increasingly widespread thanks to their intrinsic features related to formability, transparency, and removable nature which allow patients to acheive the planned teeth movement without greatly affecting their aesthetic or lifestyle [2,3]. For these reasons, nowadays clear aligners are at the basis of the so-called Clear Aligner Therapy (CAT), and they are largely employed for various malocclusion treatments and teeth misalignment defects [4,5]. Besides the Invisalign^®^ technology, thanks to the recent technological advancements in scanning, CAD/CAM software, and rapid prototyping (RP) techniques, several companies have developed their own products for dentists and orthodontists worldwide [6,7,8]. 

The biomechanical characteristics of commercial clear aligners are affected by several parameters, including their forming methods [5,9], the fitting accuracy of the aligner to the teeth [10] and, primarily, the properties of the material they are made of. Thermoplastic polymers, currently most used for the fabrication of clear aligners, are based on polyurethanes (PU) and on polyesters, such as polyethylene terephthalate glycol (PETG) [11,12]. These materials are considered biocompatible but not inert materials and are characterized by some intrinsic limitations, such as dimensional instability, low strength, and poor wear resistance [8,13]. Actually, during their use, these materials can be susceptible to mechanical actions, heat, humidity, and to the presence of foods substances together with salivary enzymes [14], leading to micro-cracks, abrasions, localized biofilm deposits, and delamination of the material. These consequences can lead to a significant loss of mechanical strength and transparency, resulting in a serious disadvantage from the patient’s perspective. Several studies are found in literature aiming to analyze the mechanical properties of clear aligners during orthodontic treatment [7,8,15,16], as well as the influence of acid/basic substances on the aesthetical functions of the aligners themselves [14,17,18,19,20]. Nevertheless, no literature work collects, in a single paper, the characterization results of most commercial clear aligners, in terms of chemico-physical features, optical properties, water absorption behavior, and color stability towards common staining agents.

Therefore, the goal of the present work is to perform a full chemico-physical characterization of most commercial clear aligners, aimed at investigating and comparing the structural, morphological, and optical features in their unaltered state and after immersion in water. Moreover, considering the undesirable and anesthetic color variations induced after the exposure of clear aligners to staining beverages [17,18,19,20], the present paper aimed also to analyze the color stability after the exposure to common staining agents and artificial saliva, in order to simulate the intra-oral aging. For these tasks, several techniques are carried out, such us attenuate total reflection Fourier infrared spectroscopy (ATR-FTIR), X-ray diffraction analyses (XRD), differential scanning calorimetry measurements (DSC), UV-visible spectrophotometry, colorimetric analyses, optical microscopy (OM), and scanning electronic microscopy (SEM). Specifically, the considered clear aligners are manufactured by different companies and the product names are Erkodur, Essix Plastic, Ghost Aligner, Zendura and Invisalign.

## 2. Materials and Methods

### 2.1. The Aligners and Their Physico-Chemical Characterization

The subjects of this investigation are five different kinds of orthodontic aligners made from thermoplastic materials. Specifically, we considered samples manufactured by different companies, characterized by the following product names: Essix ACE Plastic, Erkodur, Ghost Aligner, Zendura FLX, and Invisalign, from here called EP, EK, GA, ZN and IL samples, respectively. In Table 1 the considered aligners are summarized, also detailing the manufacturer as well as the polymer-based composition, as evidenced in the material safety data sheets.

For each brand, five thermoformed clear aligners are considered, which are initially characterized by physico-chemical analyses. Specifically, XRD and ATR-FTIR spectroscopy techniques allowed us to establish the degree of crystallinity as well as the phase composition of the considered clear aligners. XRD analyses are carried out on a rectangular plain from each sample by using a PANalytical X’PertPRO diffractometer (PANalytical Inc., Almelo, Netherlands) equipped with a Ni filter (CuKα radiation), in the exploration range 5–70 °2θ. The diffraction patterns were collected using a solid-state detector (PIXcel, PANalytical), with the detected X-ray photons binned into 0.026 °2θ constant steps and 400 s per step. The XRD patterns are elaborated by means of the Profile Fit software (HighScorePlus software package, version 4.6a, PANalytical Inc., Almelo, Netherlands), and the obtained crystalline phases are assigned by the ICDD (ICDD, Philadelphia, PA, USA) and ICSD (FIZ Karlsruhe GmbH, Eggenstein-Leopoldshafen, Germany) reference databases [21]. XRD peak broadening analysis was also performed in order to evaluate the degree of crystallinity of the considered clear aligners by measuring the full width at half maximum (FWHM) of each XRD diffraction peak. The evaluation of the degree of crystallinity can be a useful parameter, being inversely related to the transparencies of each aligner and so to aesthetical performance [22]. ATR-FTIR measurements are carried out by means of a NexusTM 870 spectrophotometer (Thermo Fisher Scientific, Waltham, MA, USA), considering a range of 500–4000 cm^−^^1^ and a resolution of 2 cm^−^^1^.

Optical properties of each aligner, in terms of transparency, are investigated by UV-vis spectrophotometer (Lambda 25 Perkin-Elmer ultraviolet/visible, PerkinElmer, Waltham, MA, USA)) by measuring the absorbance A in the visible light interval, λ = 350–750 nm. 

Both for ATR-FTIR and UV-vis measurements, the considered aligner is sectioned from canine to canine in order to expose to the beam only the labial portion. In particular, for UV-vis analysis we utilized an “ad hoc” polystyrene support in order to keep the aligner in a fixed position, exposing the same portion of sample to the visible light beam and so guaranteeing the repeatability of the measurement. 

DSC measurements (Perkin Elmer DSC 8500, PerkinElmer, Waltham, MA, USA) allow us to evaluate the thermal properties and the glass transition temperature (T_g_) of each aligner [22,23]. For this test, about 10 mg of each aligner are considered. During the measurements, the samples are heated twice under a dry nitrogen atmosphere, considering a temperature range from −70 °C to –240 °C and a heating rate of 10 °C min^−^^1^.

### 2.2. Absorption Measurement of Water, Artificial Saliva and Staining Agents

The ability of water molecules to penetrate inside the materials’ structure can be considered responsible for the decrease in mechanical cohesion [24]. For this reason, in the present paper, tests of water absorption were performed according to the International Organization for Standardization [9], working at a fixed temperature of 37 °C. In accordance with the standard ISO, the water absorption (*W_sp_*) was obtained with the help of the following equation:

$$W_{sp}=\frac{m_{2}-m_{3}}{V}$$

where *m*_2_ indicates the mass of the aligners during water immersion up to 14 days, *m*_3_ indicates the mass of the reconditioned clear aligners, while *V* represents the volume of the clear aligners. In particular, for each brand, two aligners are taken into account, and the average of the results is reported. The obtained data are expressed in μg/mm^3^.

The color stabilities of the clear aligners exposed to staining agents and to the artificial saliva, useful for reproducing intra-oral conditions, are analyzed. In particular, the considered staining agents are red wine, coffee, and nicotine, considered unfavorable in relation to the formation of severe stains on the aligner surface and causing visible color variations together with a loss of transparency [12,17]. In particular, the staining solutions are prepared as follows: (1) 120 mL of artificial saliva (Biotène Oral Balance, GlaxoSmithKline Consumer Healthcare S.p.A., Baranzate (MI), Italy) are mixed with 480 mL of deionized water; (2) undiluted red wine; (3) 3 g of coffee powder are dissolved in 100 mL of hot distilled water; (4) the nicotine solution is prepared by maintaining cigarette filters in an infusion of distilled water and then the supernatant is properly filtered. For each brand, two samples are immersed in the four staining solutions described above and maintained in immersion up to 14 days at T = 37 °C with the help of a water bath. After the immersion period, the samples are analyzed by means of the ATR-FTIR technique, UV-visible and color change analyses. The color variations of the aligners were evaluated before and after immersion both in staining agents and in artificial saliva, according to the Commission Internationale de l’Eclairage L*a*b* color system (CIE L*a*b*) [18]. In particular, the L* parameter is associated with the lightness (+ bright, − dark), while a* and b* parameters correspond to the color scale, ranging from red (+) to green (−) and yellow (+) to blue (−), respectively [19]. The values of color variations were evaluated by means of PCE Instrument (PCE Deutschland GmbH, Meschede, Germany) colorimeter before the immersion (t0) and after 7 and 14 days of immersion, respectively (t7 and t14). Before starting the measurements, the considered aligners were placed in the ultrasonic (US) bath for washing for 5 min, and then they were dried with paper. The upper central incisor of each aligner was considered for measurement, in order to guarantee a flat labial surface. To simulate the presence of a tooth behind the aligner, a light ivory plasticine was placed behind the considered incisor. Then, we performed the color measurements by keeping the optical sensor tip vertically in touch with the flat surface of the aligner. The total value of color variation (named ΔE*), corresponding to the color change before and after immersion into staining agents, is obtained by the following formula [18]:

ΔE* = [(ΔL*)^2^ + (Δa*)^2^ + (Δb*)^2^]^1/2^

The obtained data were then converted into the NBS (National Bureau of Standards) system by following the equation NBS = ΔE* × 0.92, in order to relate the color changes to a clinical standard [9]. According to the National Bureau of Standards (NBS), color variations > 1.5 are defined as noticeable, as shown in Table 2.

Moreover, the aligner as well as the immersed materials were directly examined using scanning electron microscopy (SEM, Gemini SEM 500, Zeiss, Oberkochen, Germany) analyses, useful to analyze the surface alteration related to the immersion in the staining agents. For this analysis, a small piece of each aligner corresponding to the upper incisor was properly cut and then coated with chrome in a sputter coating unit.3.

## 3. Results

### 3.1. Physico-Chemical Characterization, Optical Features, and Color Stability of the As-Received Clear Aligners

**Figure 1:**
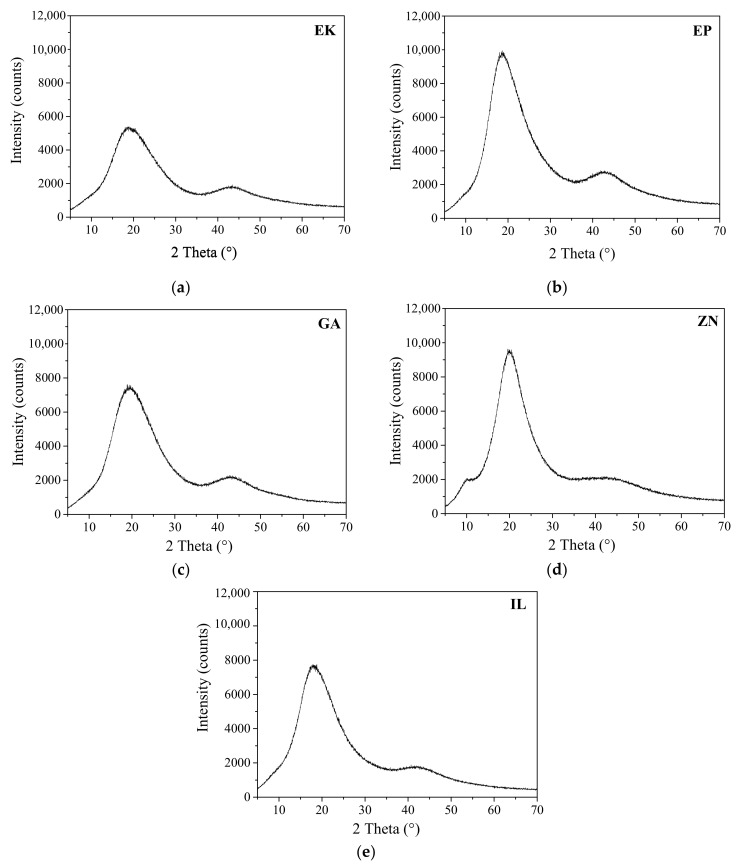
XRD patterns of the considered aligners: (**a**) Erkodur (EK); (**b**) Essix Plastic (EP); (**c**) Ghost Aligner (GA); (**d**) Zendura (ZN); (**e**) Invisalign (IL).

The XRD spectra related to the considered clear aligners are reported in Figure 1.

From the obtained data, the EP, EK, GA, and IL samples show similar diffraction patterns, characterized by two broad diffraction peaks, centered at around 19° and 43° 2θ, associable to PETG [25]. As concerns the ZN sample, it is composed of a different polymer-based, characterized by haloes at around 8°, 19° and 43° 2θ, attributable to a polyurethane [26]. In all the samples a very low crystalline order is observed. Specifically, as reported in Table 3, EK is characterized by the highest value of FWHM (of about 11° 2θ), denoting the lowest degree of crystallinity, conversely from the polyurethane-based ZN which is the most crystalline aligner, with a FWHM less than 8° 2θ.

**Figure 2:**
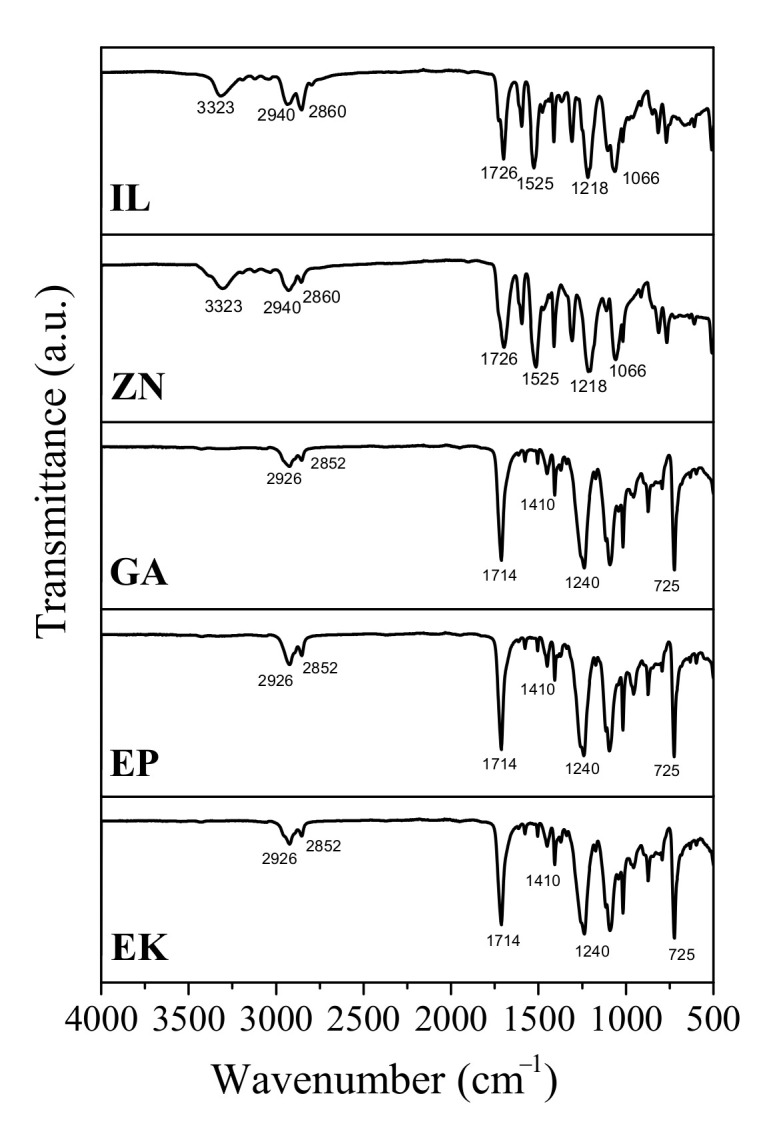
FTIR spectra of the as-received clear aligners. Erkodur (EK); Essix Plastic (EP); Ghost Aligner (GA); Zendura (ZN); Invisalign (IL).

The phase composition of the samples are confirmed by ATR-FTIR analyses, Figure 2, revealing that EP, EK, and GA have the typical infrared bands of PETG, as also reported in the literature [17]. Specifically, the band at 725 cm^−^^1^ corresponds to the out-of-plane deformation of C–H; at 1410, 1240 and 1714 cm^−^^1^, the observed bands are ascribed to CH_2_, CO-O, and C=O of ester groups, respectively; finally, the bands observed at 2852 and 2926 cm^−^^1^ can be related to the C–H symmetric and asymmetric stretching vibrations, respectively. Regarding the ZN and IL samples, the spectra show infrared bands attributable to semi rigid PU, summarized as typical bands of the N-H bond and C=O stretching vibrations at 3323 and 1726 cm^−^^1^, respectively; asymmetric and symmetric C–H at 2940 and 2860 cm^−^^1^; N–H bend vibration at 1525 cm^−^^1;^ and C=O stretching vibrations at 1700 cm^−^^1^, respectively, as typically observed in polyurethanes [17,26].

**Figure 3:**
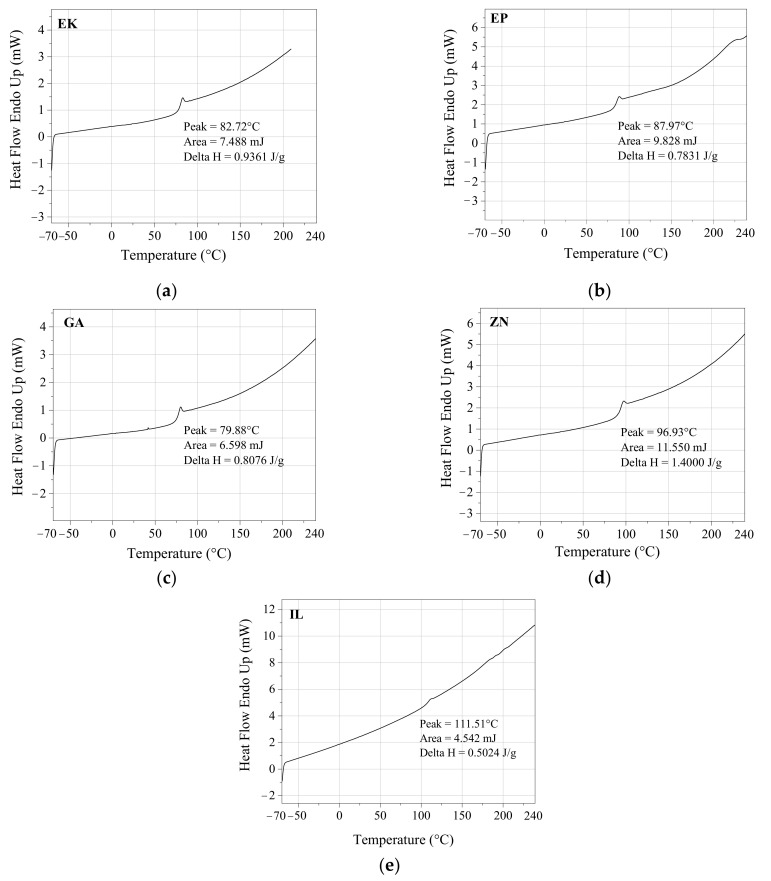
Differential scanning calorimetry (DSC) results for (**a**) Erkodur (EK); (**b**) Essix Plastic (EP); (**c**) Ghost Aligner (GA); (**d**) Zendura (ZN); (**e**) Invisalign (IL).

The DSC analyses are in agreement with the XRD and FTIR results, confirming that all the aligners are composed of amorphous thermoplastic materials, revealing a sole observable thermal phenomenon in the scanned temperature represented by the jump of the curves and corresponding to T_g_ (Figure 3). Moreover, all the aligners present a T_g_ value in the typical range observed in PETG polymers [23], corresponding to 80 °C, 83 °C, and 88 °C for GA, EK, and EP, respectively. The ZN and IL samples show higher T_g_ values, corresponding to about 97 °C and 111 °C, respectively, not typical of pure PU, but probably ascribed to the additives present in the PU-based mixtures, as also diffusely found in the literature [27,28].

**Figure 4:**
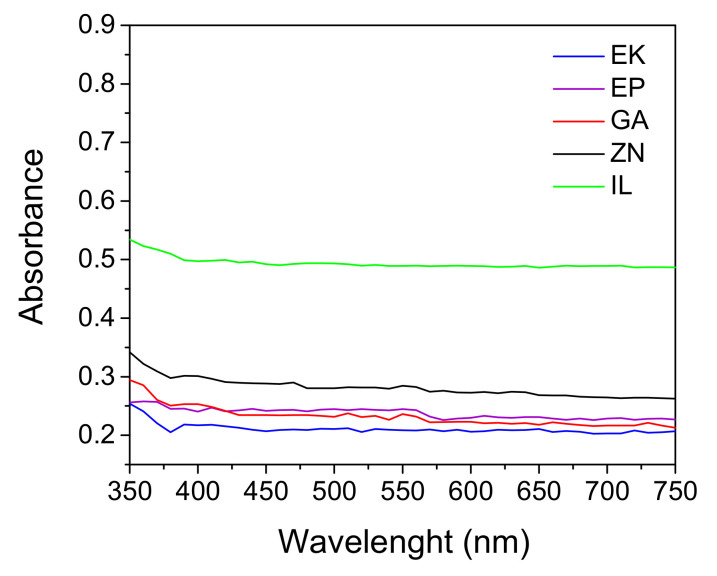
UV-visible absorbance curves of the as received clear aligners. Erkodur (EK); Essix Plastic (EP); Ghost Aligner (GA); Zendura (ZN); Invisalign (IL).

The UV-visible absorbance curves of the clear aligners are shown in Figure 4.

All the aligners present similar trends in wavelength. The EK, GA and EP samples show the lowest absorbance value in the visible range, underlining the high properties of transparency of these aligners, in agreement with the crystallinity degree (Table 3). On the contrary, IL was the least transparent sample, exhibiting the highest value of absorbency in the visible range. Despite of its amorphous structure and its FWHM value comparable with the GA and EP samples (see Table 3), the IL sample is the less transparent aligner probably due to a different thermoforming procedure or to its different chemical composition.

**Figure 5:**
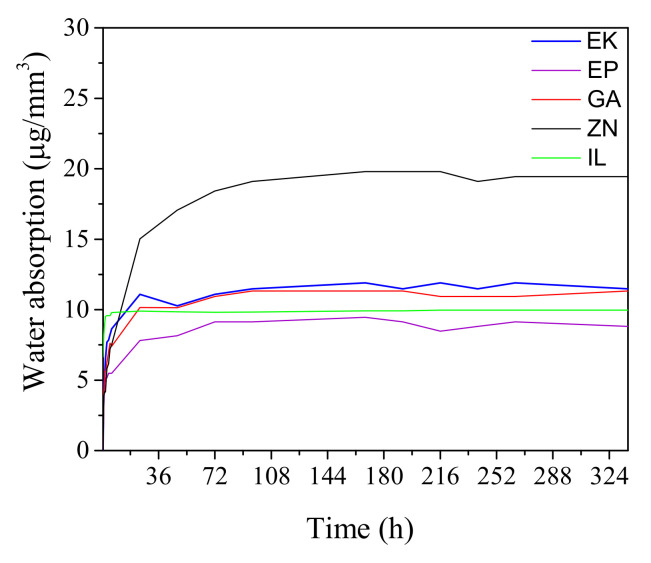
Water absorption behavior of the as-received clear aligners measured at different immersion times at a temperature of 37 °C.

The water absorption behavior of the clear aligners, measured at different immersion times, is reported in Figure 5.

After about 72 h of immersion, all the samples reached a saturation value of water absorption (W_sp_) constant until the test was over, with values ranging from 9 µg/mm^3^ to 18 µg/mm^3^, in progression from EP, IL, GA, EK, and ZN aligners.

### 3.2. Characterization Studies, Color Stability and Optical Properties of the Aligners after the Immersion into Staining Agents

**Figure 6:**
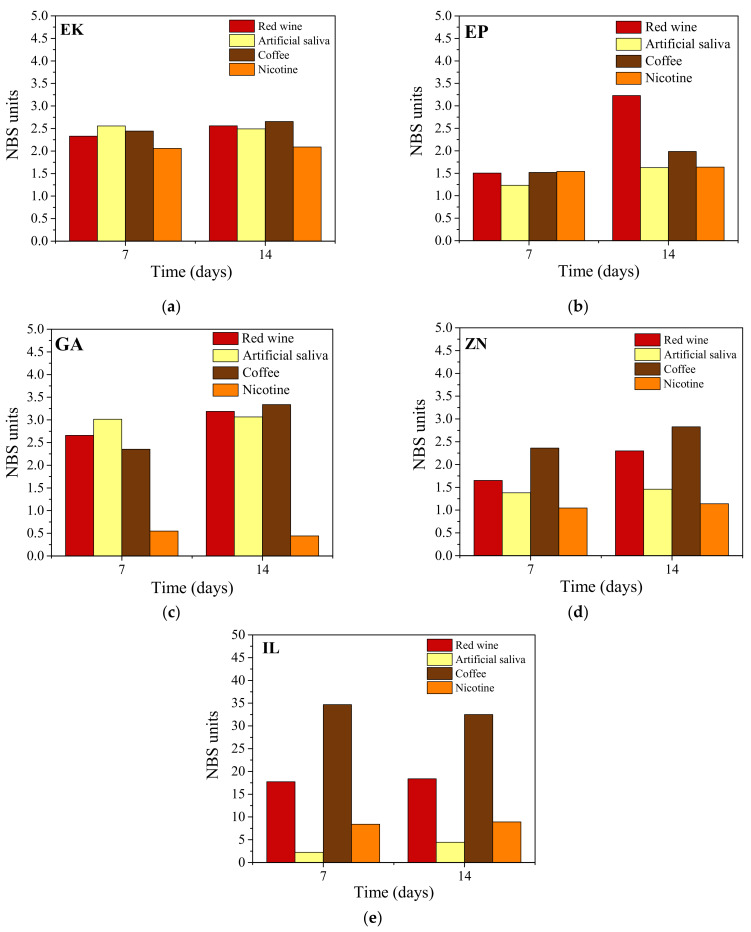
Colorimetric measurements of the considered aligners after 7 and 14 days of immersion into different staining solutions: (**a**) Erkodur (EK), (**b**) Essix Plastic (EP), (**c**) Ghost Aligner (GA), (**d**) Zendura (ZN) and (**e**) Invisalign (IL).

The color changes on the upper incisors of the clear aligners, investigated by means of colorimetric measurements, are reported in Figure 6.

In detail, independently from the considered staining solution, the EK samples show perceivable color variations already after 7 days (Figure 6a). The NBS values are almost constant up to 14 days of immersion (in the range 1.5–3.0), revealing a color change from noticeable to perceivable, as defined in Table 2.

In the case of the EP sample, (Figure 6b), after 7 days of immersion, slight changes in color are revealed for all of the staining solutions (NBS values ~ 1.5); after 14 days, coffee and nicotine provide only small increases in NBS values, while in the case of red wine, appreciable changes are recognized (NBS ≥ 3.0). In the GA samples perceivable color variations take place already after 7 days, except for the sample immersed into nicotine, which showed only extremely slight color change (Figure 6c). After 14 days, while the samples immersed into nicotine and artificial saliva remained almost constant, an increase in the color change was observed for red wine and, particularly, for coffee.

The ZN samples exhibited slight color change both after immersion into nicotine and artificial saliva solutions while, considering red wine and coffee, noticeable/perceivable change in color were observed, with NBS values of 1.5 and 2.5, respectively (Figure 6d).

When compared to the other aligners, the IL samples showed very marked color modifications, revealing even change to other color after immersion both in coffee solution and in red wine, with NBS values of 35 and 17, respectively (Figure 6e). Relatively high NBS values, from about 5 to 10, were also measured after immersion in artificial saliva and nicotine respectively.

**Figure 7:**
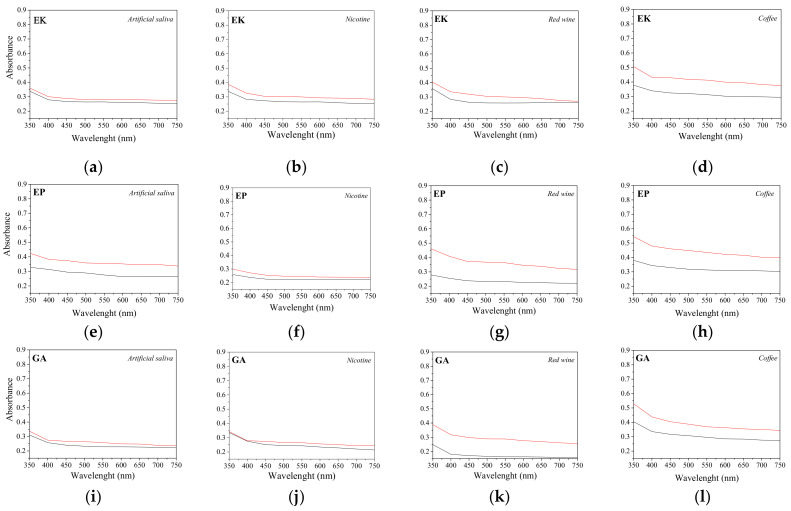

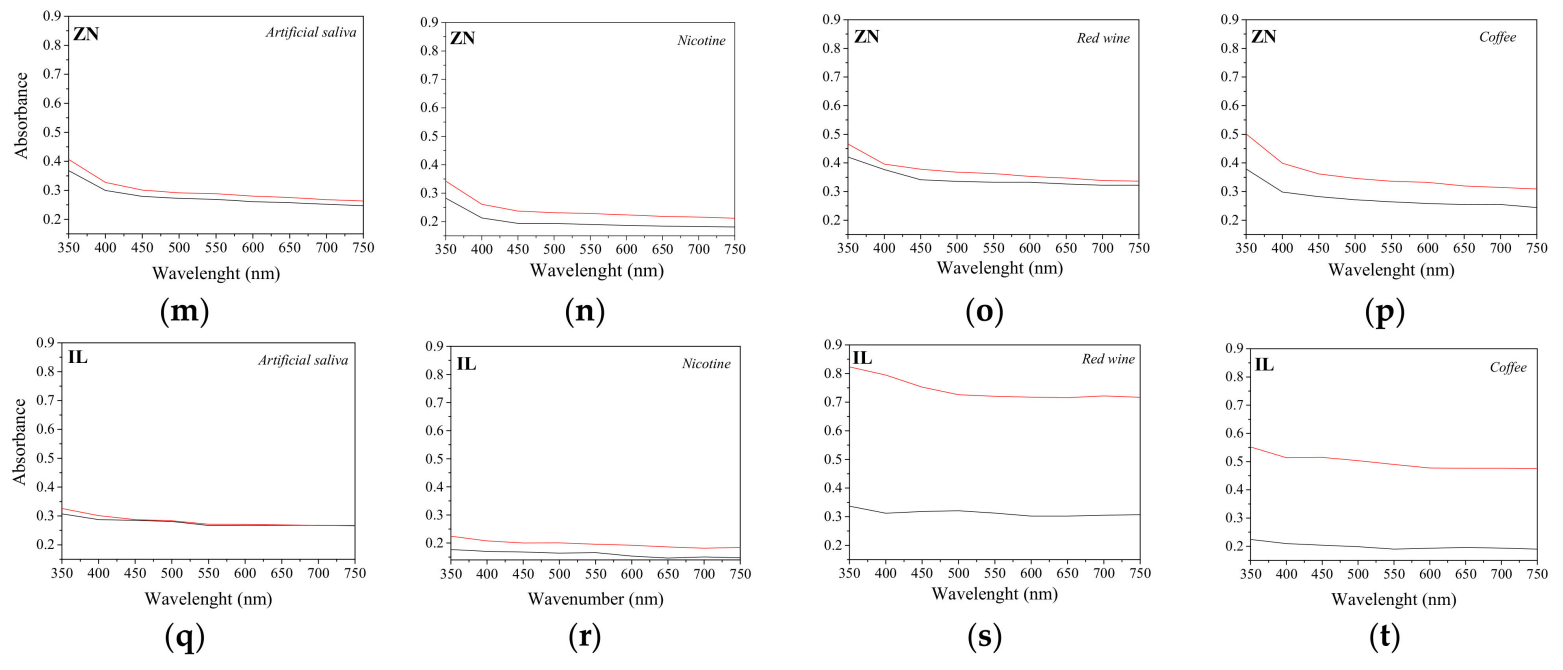
Absorbance curves of the aligners before (black line) and after (red line) 14 days of immersion in staining agents. (**a**) Erkodur (EK) in artificial saliva; (**b**) EK in nicotine; (**c**) EK in red wine; (**d**) EK in coffee; (**e**) Essix Plastic (EP) in artificial saliva; (**f**) EP in nicotine; (**g**) EP in red wine; (**h**) EP in coffee; (**i**) Ghost Aligner (GA) in artificial saliva; (**j**) GA in nicotine; (**k**) GA in red wine; (**l**) GA in coffee; (**m**) Zendura (ZN) in artificial saliva; (**n**) ZN in nicotine; (**o**) ZN in red wine; (**p**) ZN in coffee; (**q**) Invisalign (IL) in artificial saliva; (**r**) IL in nicotine; (**s**) IL in red wine; (**t**) IL in coffee.

The UV-vis analyses, performed on the upper incisors before and after 14 days of immersion into staining solutions, are shown in Figure 7. The graphs are represented using the same y-scale in order to compare the behavior of the aligners characterized by different brands and immersed in different solutions. All the samples presented an increase in the absorbance curves, underlining the loss of transparency following the interaction with the staining agents. In particular, the PETG-based samples (EK, EP, and GA) show comparable loss of transparency, with an increase in absorbance value up to 30% along all the wavelengths after immersion into red wine and coffee. As concerns the PU-based sample (ZN), smaller increases in the absorbance values were observed while, according to the colorimetric measurements, the IL aligner showed the highest enhancement of absorbance values, up to 60% after immersion both into red wine and in coffee solutions.

**Figure 8:**
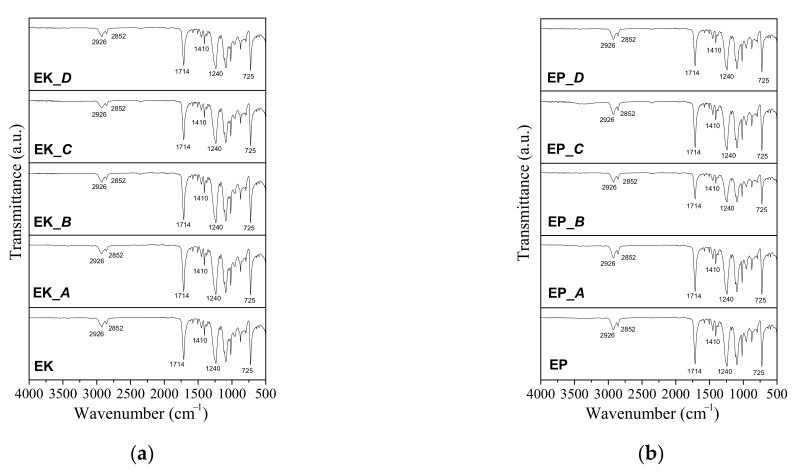

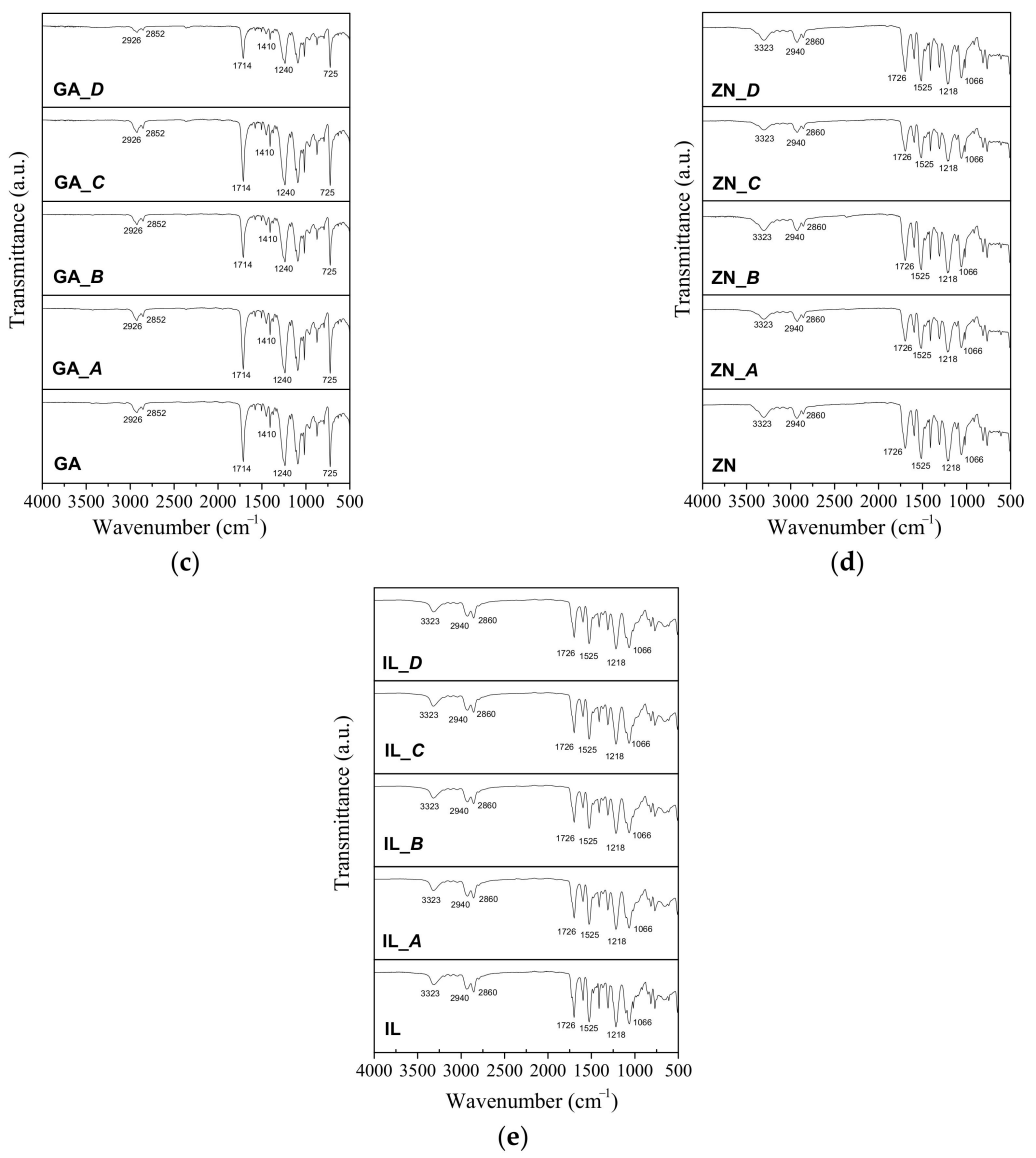
Comparison between the FTIR spectra of the as-received aligners and those immersed for 14 days into staining agents. (**a**) Erkodur as received (EK) and after exposure to artificial saliva (EK_*A*), nicotine (EK_*B*), red wine (EK_*C*) and coffee (EK_*D*); (**b**) Essix Plastic as received (EP) and after exposure to artificial saliva (EP_*A*), nicotine (EP_*B*), red wine (EP_*C*) and coffee (EP_*D*); (**c**) Ghost Aligner as received (GA) and after exposure to artificial saliva (GA_*A*), nicotine (GA_*B*), red wine (GA_*C*) and coffee (GA_*D*); (**d**) Zendura as received (ZN) and after exposure to artificial saliva (ZN_*A*), nicotine (ZN_*B*), red wine (ZN_*C*) and coffee (ZN_*D*); (**e**) Invisalign as received (IL) and after exposure to artificial saliva (IL_*A*), nicotine (IL_*B*), red wine (IL_*C*) and coffee (IL_*D*).

Concerning the ATR-FTIR measurements performed after the immersion in staining agents (Figure 8), for all the aligners the spectra appear almost overlapped with those referred to as as-received samples. The obtained results denote that, within the instrumental sensitivity, the polymeric bonds are not strongly affected by the organic substances present in the staining solutions.

**Figure 9:**
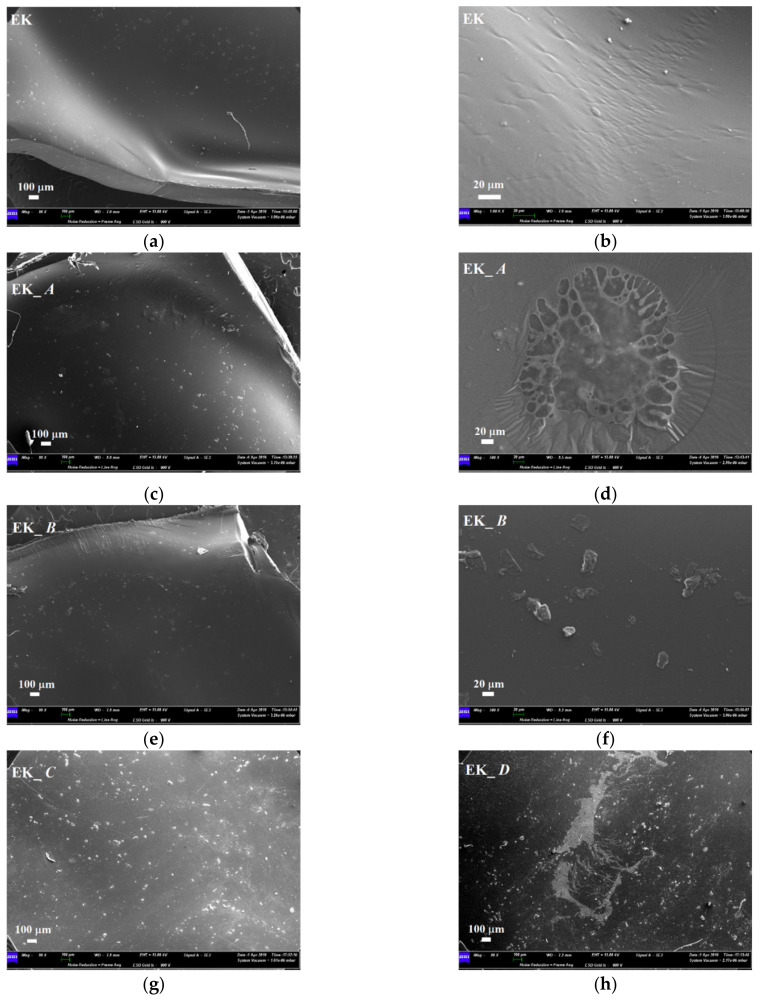
Comparison between SEM micrographs of Erkodur (EK) aligner before and after 2 weeks of immersion into staining beverages; (**a**,**b**) as-received sample; (**c**,**d**) sample immersed into artificial saliva (A); (**e**,**f**) sample immersed into nicotine (B); (**g**) sample immersed into red wine (C); (**h**) sample immersed into coffee (D).

SEM micrographs of the EK samples, before and after 14 days of immersion in staining agents, are reported in Figure 9. Before the immersion, the EK surface appeared quite smooth with some impurities attributable to superficial deposits (Figure 9a). At higher magnification, the presence of superficial ripples, probably related to mechanical material deformation following the thermoforming process, were observed too (Figure 9b). After immersion in the artificial saliva, more deposits were visible, some of them in the form of bubbles, probably attributable to the artificial saliva exploding under the electron beam (Figure 9c,d). The micrographs in Figure 9e,f show the aligner surface after immersion into nicotine solution wherein sporadic and not uniformly distributed impurities, with different morphology and dimensions, are recognizable. The surface of the sample immersed in red wine appeared covered by several impurities, becoming glossy under the electron beam (Figure 9g). As reported in the literature [26], these deposits can be attributed to the wine entering the polymeric material and creating a non-homogeneous phase dispersed inside the polymer itself. Similar results were observed after immersion in the coffee solution, together with the presence of deposits characterized by an irregular morphology (Figure 9h).

The others PETG-based aligners (EP and GA), reveal similar behavior after immersion into staining agents, with respect to the EK sample. In particular, before immersion, the samples showed some regions characterized by a more irregular surface compared to EK, with small cavities and more impurity residues. After immersion into artificial saliva, only small bubbles were shown, while the surface of the EP and GA samples, immersed into nicotine solution, revealed the presence of deposits as well as of sporadic microbial growth. Finally, the formation of localized and homogenously dispersed impurities was recognizable after immersion into red wine.

**Figure 10:**
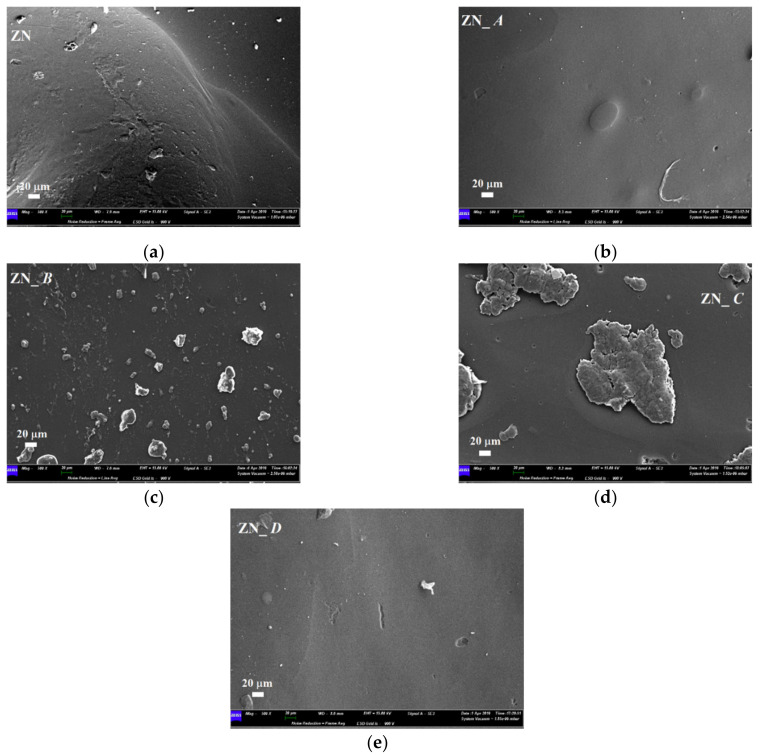
Comparison between SEM micrographs of Zendura (ZN) aligner before and after 2 weeks of immersion into staining beverages; (**a**) as-received sample; (**b**) sample immersed into artificial saliva (A); (**c**) sample immersed into nicotine (B); (**d**) sample immersed into red wine (C); (**e**) sample immersed into coffee (D).

Regarding the surface of PU-based aligner (ZN) before the immersion, the presence of several cavities and impurities was revealed (Figure 10a), and the surface remained almost unaltered when artificial saliva and coffee solutions were considered (Figure 10b,e). Contradictorily, in the case of the nicotine solution, several microbial growths appeared on the sample surface (Figure 10c), while immersion in red wine led to the formation of several superficial deposits (Figure 10d).

**Figure 11:**
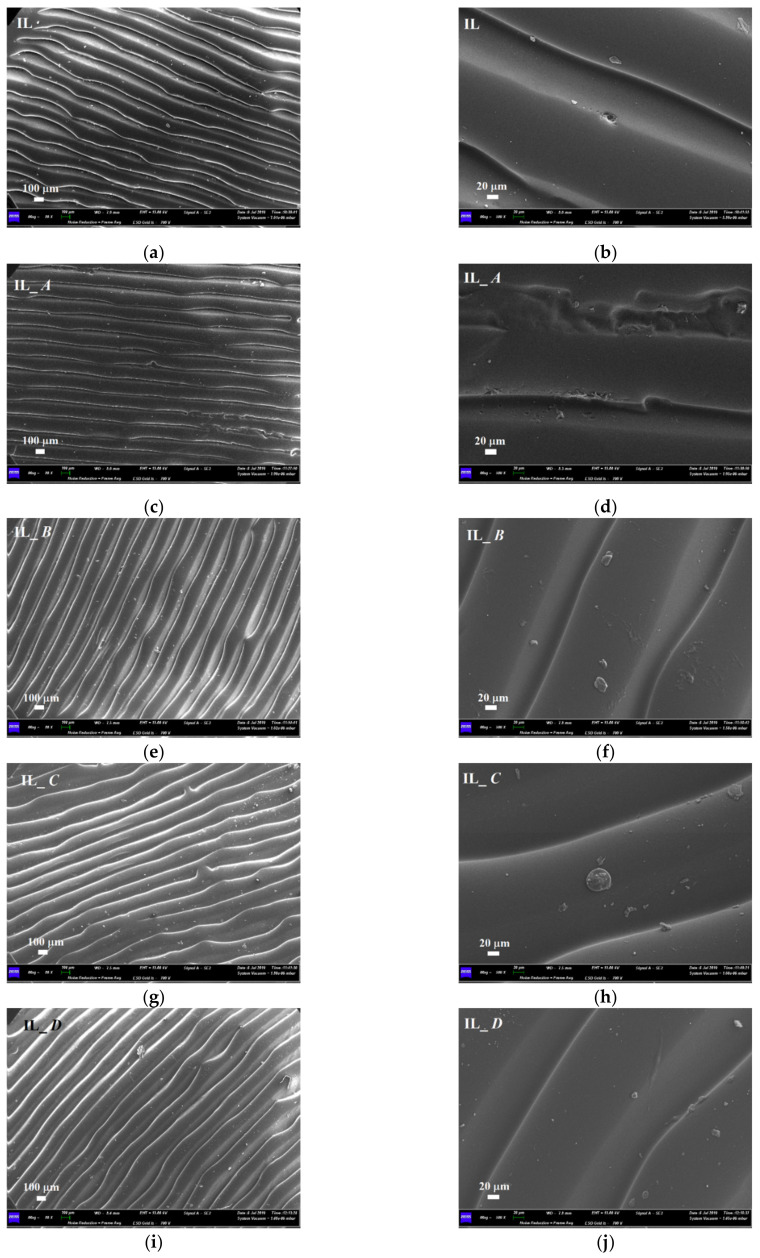
Comparison between SEM micrographs of Invisalign (IL) aligner before and after 2 weeks of immersion into staining beverages; (**a**,**b**) as-received sample; (**c**,**d**) sample immersed into artificial saliva (A); (**e**,**f**) sample immersed into nicotine (B); (**g**,**h**) sample immersed into red wine (C); (**i**,**j**) sample immersed into coffee (D).

Finally, SEM images of the IL aligner are shown in Figure 11. Before immersion, the sample surface appeared very different with respect to the other brands of aligners, and it was mainly composed of regular and homogeneously distributed wrinkles, practically unaltered after the immersion into artificial saliva (Figure 11a–d). On the contrary, after immersion into the other staining agents, several pseudo-spherical contaminations could be observed, as shown in Figure 11f,h,j.

## 4. Discussion

The chemico-physical analyses (XRD, FTIR and DSC) reveal that some of the as received aligners (EP, EK, GA) are composed of PETG-based thermoplastic materials [17,23,25], while ZN shows a different polymer base, attributable to polyurethane (PU) [26,27,28]. However, all the aligners are characterized by very low crystalline order, as underlined by measuring the full width at half maximum (FWHM) of each XRD diffraction peak. In particular, the EK sample reveals the lowest degree of crystallinity, being characterized by the highest value of FWHM, contrary to the polyurethane-based ZN which appears to be the sample with the highest crystalline order (lowest value of FWHM). Considering that the degree of crystallinity is inversely related to transparency [22], the analysis of the FWHM parameter should be considered an important rapid check to control the aesthetical performances of the aligners themselves.

In agreement with the crystallinity degree, the highest transparency was that of the EK sample, followed by the others PETG-based aligners, GA and EP, which was confirmed by means of UV-visible measurements. In particular, the EK, GA, and EP samples show the lowest absorbance values in the visible range while IL, despite of its amorphous structure and its FWHM value comparable with GA and EP, appears to be the least transparent aligner, probably due to a different thermoforming procedure or to its different chemical composition.

The water absorption investigations furnish an important indication of the interaction of the samples with the aqueous external environment and of their ability to maintain their characteristics during usage, thereby preventing mechanical degradation phenomena [10,11,12,14]. The water molecules’ penetration into the material’s structure, occurring in oral environment, can lead to changes in the aligner from a physicochemical point of view, causing mechanical degradation and swelling phenomena. For these reasons, as also reported in the literature [11], the ideal orthodontic clear aligner must have low water absorption properties, similar to what was observed for EP, while the worst performance was given by the PU-based aligner.

The clear aligners, in relation to the considered brands, show different interaction behavior when immersed in the staining solutions [12,17]. Most aligners revealed color variations already after 7 days of immersion, denoting that the polymers tend to rapidly interact with the external environment, as was also observed in the water absorption measurements. In particular, only slight changes in color were observed for ZN, EK, EP, and GA aligners after 7 days of exposure in all of the considered staining solutions, while after 14 days, particularly in the case of red wine and coffee, an increase in the color change was noted [18]. The most appreciable variations in color were obtained from the IL samples, exhibiting relatively high NBS values after immersion in artificial saliva and nicotine, and even changes towards other colors after immersion both in coffee solution and in red wine. This result can probably be attributed to the thermoforming process of the IL sample, leading, differently from the other brands of aligners, to the formation of a wrinkled surface able to entrap impurity residues and deposits, as confirmed by SEM observations.

## 5. Conclusions

The present paper focuses on a direct comparison between commercial orthodontic aligners used for Clear Aligner Therapy, analyzing them in terms of chemico-physical features, optical properties, behavior toward water, and color stability after immersion into common staining agents and artificial saliva. Five thermoformed clear aligners, manufactured by different companies, are considered; three of them are made of PETG (Erkodur, Essix Plastic and Ghost Aligner), one is a PU-based polymer (Zendura), while the last one is composed by a mixture of PU and PETG (Invisalign), as confirmed by XRD, FTIR-ATR, and DSC analyses. From XRD, all the samples reveal a low crystallinity degree, particularly marked for the PETG-based materials, appearing also characterized by the highest properties in terms of transparency in the visible range, as confirmed by UV-visible absorption spectra.

From the study of water absorption, which is an important control parameter to predict unavoidable mechanical degradation during usage, we measured different behavior between the clear aligners, with the worse performance being given by the PU-based aligner.

Regarding the color variations of the aligners after immersion into staining agents, evaluated by colorimetric measurements, all the considered solutions contributed to alter the aesthetical features of the samples, particularly when the aligners were immersed into coffee or red wine. In addition to the color variation, after immersion all the aligners revealed an increase in the absorbance curves and so a loss of transparency. The most appreciable variations in color were obtained in the IL samples followed by GA, while only noticeable changes were attributed to the ZN, EK and EP samples. In particular, the IL sample exhibited even changes towards other colors, probably due to the thermoforming process which lead to the formation of a wrinkled surface able to entrap impurity residues, as confirmed by SEM observations.

## Figures and Tables

**Table 1:** The considered clear aligners in terms of manufacturer and polymer-based composition, as evidenced in the material safety data sheets.

Samples Denomination	Product Name (Manufacturer)	Polymer-Based Composition
EP	Essix ACE Plastic (Dentsply Sirona, York, PA, USA)	PET
EK	Erkodur (Erkodent Erich Kopp GmbH, Pfalzgrafenweiler, Deutschland)	PET
GA	Ghost Aligner (BART MEDICAL S.r.l., Mezzano, Italy)	PET
ZN	Zendura FLX (Zendura, Bay Materials LLC, Fremont, CA, USA)	Polyurethane (PU) system
IL	Invisalign (Align Technology, Inc. San Jose, CA, USA)	Mostly PU

**Table 2:** National Bureau of Standards (NBS) units. Description of color variations.

**National Bureau of Standards Units**	**Descriptions of Color Changes**	NBS = 0.92 × ΔE*
0.0–0.5	Trace. Extremely slight change	
0.5–1.5	Slight. Slight change	
1.5–3.0	Noticeable. Perceivable	
3.0–6.0	Appreciable. Marked change	
6.0–12.0	Much. Extremely marked change	
12.0 or more	Very much. Change to other color	

**Table 3:** FWHM (full width at half maximum) values of the clear aligners.

	EK Aligner	EP Aligner	GA Aligner	ZN Aligner	IL Aligner
Full Width at half maximum (FWHM) (°2θ)	11.315	9.188	10.345	7.727	9.833
